# Beyond Grade Point Averages and Medical College Admission Test Scores: A Thematic Analysis of Exceptional Performing Medical Student Applications

**DOI:** 10.15694/mep.2021.000151.1

**Published:** 2021-06-02

**Authors:** Matthew Pflipsen, Dario Torre, Steven Durning

**Affiliations:** 1 Uniformed Services University of the Health Sciences

**Keywords:** medical school, student selection, academic performance

## Abstract

This article was migrated. The article was marked as recommended.

**Introduction:** Medical school admissions committees are tasked with selecting the best students for their institution and historically rely on grade point averages and Medical College Admission Test scores as measures for academic success. Yet research and expertise theory suggest that personal characteristics play a critical role in exceptional performance. Understanding the characteristics of exceptional performing medical students upon application to medical school could contribute to the holistic review process and selection decisions of medical universities.

**Methods:** The purpose of this study was to identify themes in the American Medical College Application Service (AMCAS) application that reflect the characteristics of exceptional performing medical students when they applied to medical school. The authors completed an inductive thematic analysis of the primary AMCAS application of exceptional performing medical students. Selection to both Alpha Omega Alpha Honor Medical Society and the Gold Humanism Honor Society defined exceptional performance.

**Results/Analysis:** 22 (4.5%) of 485 medical school graduates between 2017 and 2019 met criteria for exceptional performance. The authors identified seven themes from the AMCAS applications: success in a practiced activity, altruism, entrepreneurship, passion, perseverance, teamwork, and wisdom.

**Discussion:** The seven identified themes were consistent with the personal characteristics associated with both expertise theory and the AAMC’s core personal competencies for medical student success. By constructing an understanding of the personal characteristics exceptional student performers display in their applications to medical school, these themes offer an additional lens for medical school admission committees to assess a student’s potential to be successful in medical school.

## Introduction

Medical school admission committees have often used prior academic performance as a proxy for future exceptional performance in medical school through a reliance on undergraduate grade point average (uGPA) and Medical College Admission Test (MCAT) scores ( Monroe *et al.,* 2013). Yet, while MCAT scores and uGPA have been shown to predict success in the pre-clinical years of medical school ( Donnon, Paolucci and Violato, 2007; Busche *et al.,* 2020), they correlate less well with performance in the clinical years and beyond ( White, Dey and Fantone, 2009; Haight *et al.,* 2012; Saguil *et al.,* 2015). Instead, personal competencies become valued during these clinical years and likely play a significant role in the ability of clerkship students and residents to perform successfully ( Lievens, Ones and Dilchert, 2009; Haight *et al.,* 2012; Hojat, Erdmann and Gonnella, 2013).

Developing a better understanding of what characteristics exceptionally performing medical students possess upon entrance to medical school is necessary if we seek to optimize the recruitment of students with the best potential to be exceptional performing physicians. As a medical student, top characteristics that faculty consider when assigning an honors clerkship grade are curiosity, dependability, taking ownership, and high ethical standards ( Herrera *et al.,* 2019); while top characteristics valued in first year residents are responsibility, teamwork, empathy, and prioritization ( Furstenberg and Harendza, 2017). Other research demonstrates that conscientiousness, extraversion, and grit are likely associated with successful clinical performance ( Haight *et al.,* 2012; Miller-Matero *et al.,* 2018; Cortez *et al.,* 2019). The Association of American Medical Colleges (AAMC) also recognizes the value of personal characteristics and describes nine core competencies for entering medical school students to be successful ( Association of American Medical Colleges, (2021a); Koenig *et al.,* 2013).

While this previous research does enlighten us to the personal characteristics of medical students that are likely to perform well, research to date has not determined what characteristics are actually exhibited by exceptional performing medical students when they applied to medical school. Koenig *et al.* advocate for future study of potential tools to evaluate personal competencies for entering medical students and suggest accomplishment records as a tool for further research ( Koenig *et al.,* 2013). The American Medical College Application Service (AMCAS) application offers such a tool by allowing applicants to describe up to 15 work and activity experiences. The three most meaningful experiences offer additional narrative for applicants to reflect on “the personal growth [they] experienced as a result of [their] participation” ( Association of American Medical Colleges, (2021b)). Understanding which characteristics exceptional performing medical students exhibit when applying could help admission committees optimize their decisions regarding matriculation and challenges the premise that admissions committees must continue to heavily weigh the traditional measures of uGPAs and MCAT scores to ensure academic success in medical school.

Deliberate practice is a theory that also explains the acquisition of expert performance. Within this theory, innate abilities, such as intelligence, are not essential for exceptional performance. Rather, expert performance is the result of deliberate (or effortful) practice ( Ericsson, 2015). In relation to medical student applicants, the theory would support that expert performance in academics and medicine is not rooted simply in measures of intelligence, but instead, is the result of engagement of deliberate practice within study habits ( Moulaert *et al.,* 2004; Plant *et al.,* 2005; Duvivier *et al.,* 2011). Thus, deliberate practice suggests that exceptional performance in medical school depends on more than uGPAs and MCAT scores and can provide a lens through which to interpret our research.

This is the first qualitative study to our knowledge that explores the characteristics of exceptional performing medical students within primary medical school applications using applicants’ own words to capture findings. The purpose of this study is to describe these characteristics by answering the following research question: Which characteristics in medical student AMCAS applications are associated with exceptional performing medical students when they applied to medical school?

## Methods

Every medical school applicant submits an AMCAS application for medical school admission within the United States (US). The AMCAS application provides a consistent standard that can be generalized to all US medical schools and allows for interpretation of data derived directly from the information provided by the applicant. The work and activity section provides up to fifteen experiences across the domains of teaching, community service, research, employment, recognition, extracurricular activities, and athletics. Three of these experiences can be designated as most meaningful, where applicants can elaborate on the “transformative nature of the experience” and “the impact [the applicant] made while engaging in the activity” ( Association of American Medical Colleges, 2021b). We focused on these sections because we could best capture themes within the rich descriptions written directly by the matriculate. We did not include application elements such as letters of recommendation (LOR), interview comments, and admission committee member comments. While these supplementary documents can provide additional information on the characteristics of the applicants, they either give us a secondary interpretation of the applicant through another person’s viewpoint (LORs, interviews, and admission committee comments) or the formats may not be consistent across medical school admissions offices (interviews).

We explored these applications from a constructivist paradigm using thematic analysis. Thematic analysis is a robust method to use when seeking to understand a set of experiences across a data set ( Boyatzis, 1998; Braun and Clarke, 2006; Kiger and Varpio, 2020). The multidisciplinary nature of our research team provided a variety of perspectives to approach our research question. The first author (MP) is an accomplished clinician educator with firsthand experience in leadership roles in the military and medical education. The other two co-authors (SD, DT) have extensive experience both serving on medical school admission committees and performing research on medical student performance.

We also obtained demographic data, MCAT scores, and uGPAs from each student’s AMCAS application. These data describe the composition of the exceptional performing medical students in our sample via traditional admission measures. The study was approved by the Uniformed Services University of the Health Sciences (USU) Institutional Review Board (USUHS.2020-042).

### Subjects

Our unit of analysis was exceptional performing medical students from three consecutive graduating classes (2017-2019) at the USU. We defined exceptional performing medical students as students elected to both the Alpha Omega Alpha Honor Medical Society (AΩA) and the Gold Humanism Honor Society (GHHS). The mission of the GHHS is “to recognize individuals who are exemplars of humanistic patient care and who will serve as role models, mentors, and leaders in medicine” ( Arnold P. Gold Foundation, (2021a, p2), while the mission of AΩA is to “improve care for all by recognizing high educational achievement, encouraging the development of academic and community leaders, and promoting service to others” (Alpha Omega Alpha Honor Medical Society, (2021, p4).To be elected to both, students would be expected to display not only high scholastic achievement, but also professionalism, leadership, community service, compassion and humanistic clinical care.

At the USU, the top 25% of academic performers are invited to submit an application for AΩA; while consideration for GHHS is through a peer nominated process solicited during the students’ core clerkship year. The selection process for each society is distinct and separate ( Arnold P. Gold Foundation, (2021a); Alpha Omega Alpha Honor Medical Society, (2021)) and induction for each is restricted to 15% of the medical school class. With 160 chapters for either AΩA or GHHS ( Arnold P. Gold Foundation, (2021b); Alpha Omega Alpha Honor Medical Society, (2021)), using election to both societies serves as a feasible proxy of exceptional performance at other medical schools in the United States.

### Data analysis

We performed descriptive analysis on demographic variables, MCAT scores, and uGPA using Excel 2016 for Windows (Microsoft, Redmond, Washington). For our thematic analysis, we took an inductive approach and performed iterative cycles of investigation by year group. The unit of coding were student statements within the work/activities and personal essay sections of each AMCAS application. Following the methods described by Boyatzis ( Boyatzis, 1998) and outlined in a 6 step process by Braun and Clarke ( Braun and Clarke, 2006), one researcher (MP) generated initial codes after familiarizing himself with the data from the 2017 year group. These initial codes were analyzed and compared to search for themes that describe the characteristics of the students and then shared with the other two researchers (SD, DT) who reviewed the data, codes, and initial themes. Through an active and interpretive process, all three researchers discussed, analyzed, compared, and mapped relationships among the data and codes to reach complete consensus on the definition and naming of all constructed themes. One researcher (MP) continued to code the applications from the 2018 and 2019 year groups, applying the initially constructed themes and generating new codes until the following criteria were fulfilled: the data no longer yielded any further themes, the constructed themes managed the new data without requiring further modifications (theoretical sufficiency) ( Varpio *et al.,* 2017), and all applications had been reviewed. These final data, codes, and themes were also independently reviewed and analyzed by SD and DT until we reached complete consensus on all constructed themes. All AMCAS applications were coded within NVIVO 12.0 for macOS (QSR International, Burlington, Massachusetts).

## Results/Analysis

Students inducted into both AΩA and GHHS represented 4.5% (n = 22) of all medical school graduates (n = 485) from 2017 to 2019 at the Uniformed Services University. The average age at matriculation was 26 (SD 4.5, range 22-40) and 40% (n = 9) were female gender. One third (n = 7) had prior military experience either on Active Duty, in the Reserves, or in the National Guard. Approximately half (n = 10) took the MCAT more than once and the average of their highest MCAT score was 31.45 (SD 2.3, range 27-36). The mean cumulative grade point average was 3.59 (SD 0.31, range 2.71-4.0).

We coded statements within each experience and personal essay of all 22 exceptional performer’s AMCAS applications, representing 175 pages of examined text. Each statement could be coded in more than one theme. Theoretical sufficiency was reached after reviewing 15 applications. Seven themes were constructed from the data: success in a practiced activity, altruism, entrepreneurship, passion, perseverance, teamwork, and wisdom ( Table 1).

### Success in a practiced activity

This theme was characterized by demonstrating ongoing practice and dedicated engagement in an activity which led to an improvement in one’s ability or resulted in success. The types of activities varied and included training, participation in internships, formal practice, and work. Success was mostly displayed through either high achievement in competition, recognition for performance, or a promotion to a higher position. When not associated with a concrete measure of success, dedication to the activity resulted in significant improvement in an ability or skill. Commitment to and/or extended practice of the skill was evident across this theme, often with the individual dedicating numerous hours to the activity:

*I participated in an intensive summer training program to develop the skills and knowledge needed to achieve significant gains in student achievement while teaching... I completed my two year [Teach For America] commitment teaching biology... I was awarded the [annual] Outstanding Educator of the Year award by the student body. (006)*

### Altruism

This theme was characterized by a genuine desire to selflessly help an individual or community and reflects an internal reward the student feels from their experience. Characteristics demonstrated within altruism were selfless service, compassion, and generosity. Most exceptional performing medical students committed to participating in activities that directly impacted, served, assisted, or improved others, often benefiting disadvantaged, underserved, or those with disabilities. Often the experience gave a feeling of deep satisfaction and personal fulfillment:

*Each week I meet and interact with children with developmental disabilities and various nervous system traumas, and I am able to see the progress and improvements that they make each week... It also brings me immense joy to make the children laugh and smile, and for a few brief moments, forget about their ailments. (008)*

This theme also includes activities such as mentoring, teaching, or coaching other groups of individuals, reflecting a desire to help others improve:

*Whether it is through training others, explaining the insulin-glucagon axis, or teaching the biomechanics of knee position in a squat, I find a deep satisfaction in breaking down complex concepts in a meaningful way for others and helping them to achieve a healthy and fulfilling life. (022)*

A simple statement of volunteering or engaging in an activity by itself did not meet the definition of this theme: *‘[I] mentored four freshman students through the challenges of Plebe Year.’ (019)* Instead, there must be an understood desire and commitment by the student to help that is associated with evidence of a direct impact on others.

### Entrepreneurship

This theme was defined as taking initiative to accomplish or create something. There was often a motivation by a personal desire or concern for doing something innovative. Engagement in the activity was either initiated on one’s own or collaboratively with another person:

*A roommate of mine happened to work for someone washing windows, just as I had. We decided to start our own business and take out a loan for supplies. We paid off the loan in a matter of weeks and continue to wash together today... (018)*

Entrepreneurship also involved initiative in developing anew process with an explicit goal to improve a system or fix a problem:

*What I inherited with that office was a mess. While the organization was solid, its finances were not. In fact the chapter was in debt... I was able to resolve the debt, formulate a functional budget, and secure a reasonable financial future the fraternity. (017)*

Routine responsibilities of a leadership position were not considered as entrepreneurship as these responsibilities represent routine requirements of a position: *‘as the baseball representative for the program [I] was responsible for developing proactive strategies to increase awareness of substance abuse’ (010)* Entrepreneurship was also not established through being a follower or under the direct supervision of someone else: *‘As the student leader of the project, I began to help him [professor] with his efforts...’ (006)*

### Perseverance

This theme was characterized by an ability to overcome hardship, adversity, difficulty, or setback through hard work, commitment, or determination. There was often a struggle or challenge that required resiliency, flexibility, or resourcefulness in order to adapt and endure:

*If a patient requiring therapy had limited access because of an insurance company, I wrote letters demonstrating gains to justify continued care... When patients needed medical equipment, I called their physician to validate the need. (005)*

Usually the end result of persevering was a positive outcome, although in some situations there was an increase in confidence in the individual:

*Amidst doors slamming in my face and being made fun of, I pressed onward in a cause I believed in. I learned that my happiness stemmed from honest and dedicated hard work. As I worked through adversity I found confidence. (018)*

### Passion

This theme was characterized by an intense interest for an activity or pursuit. The theme was often identified at the latent level, reflecting that passion was likely an underlying phenomenon expressed across experiences; although it also would manifest through a commitment to a single experience. In both cases, there was a dedication and engagement that was exhibited consistently over time. Enthusiasm, enjoyment, aspiration, and intrigue were often expressed when describing the experiences:

*My high school independent study (...) evolved into a passion and immersion into the Duchenne muscular dystrophy(DMD) community, including an appearance on Good Morning America, conferences, house calls, and disheartening deaths of friends with DMD... Throughout the academic years, I developed my thesis project (...) to elucidate post-transcriptional differences in DMD pathology. While volunteering as a counselor at Muscular Dystrophy Association summer camps for three years... (007)*

A standalone statement about being passionate for something, such as *‘I am passionate about the work that I do, and I enjoy public speaking’ (008)* or *‘...a passion rather than a gift for the sciences led me to pursue a pre-medical neuroscience major’ (006),* did not rise to the level of meeting the definition of this theme.

### Wisdom

This theme was described by gaining new insight, self-discovery, or awareness from an experience or activity, resulting in an examination of an internal thought process that leads to personal growth. Most exceptional performers reflected on a personal situation in which actions and feelings were analyzed or evaluated, leading to a transformative process where the student learned through and from their experience:

*This experience taught me the importance of fully investing myselfin whatever I am involved in. Genuine care for the kids brought me success, not the pursuit of success. (018)*

Insight often derived from experiences that related to leadership, empathy, tolerance, or self-care. The following example demonstrates a reflection on cultural bias and an understanding of being tolerant of differences:

*From this experience I learned the importance of avoiding cultural biases and being culturally aware. Integrating this lesson into my lifehas enriched my professional and personal relationships by allowing me to draw upon similarities, rather than differences (020)*

while this applicant shows insight gained from a leadership experience:

*The challenge has always been getting my Marines to want to accomplish what they wouldn’t otherwise... The highs include... the benefits of truly influencing a young man for the better, whether professionally or personally. The lows have been more instructive as enforcing standards is rarely easy and the task of correcting someone in the best manner has only come with experience. (022)*

There were statements about what it was like to be something or someone: *‘[I] began to appreciate the dedication demands and day-to-day responsibilities of a doctor’ (010),* or a gaining of a better understanding about a process or system: *‘I learned much about national regulations that stipulate a doctors’ care’ (002),* or being inspired from an experience: *‘I realized that the personal side of medicine appealed to me’ (016),* but these statements did not demonstrate the self-discovery or personal growth associated with this theme.

### Teamwork

This theme was defined as an appreciation for or reflection of the value of teamwork, collaboration, interpersonal relationships and/or camaraderie. Exceptional performers either demonstrated the value in working together with others to achieve goals and be successful:

*Being part of the program allows me to spend the majority of my time outside of class with like-minded individuals, working together to help each other achieve our goals so that we may all become successful officers upon commissioning. (016)*

or placed an emphasis on teamwork over individual effort or achievement:

*While playing rugby, I have learned that a strong team effort can achieve far more than any individual can. (008)*

When building camaraderie, there was a cultivation of relationships and a development of a bond with others, as in the following example:

*Having led them and the others through almost 200 combat patrols, having eaten, slept, and fought with them for months, our unbreakable bond of brotherhoodwas one borne of the crucible of war. We were a family, and their injuries affected me no differently than if we were blood relatives. (019)*

**Table 1.** Themes and Representative Quotes from Exceptional Performing Medical Students, USU 2017-2019

Theme	Representative Quotes
Success in a practiced activity	*‘I began at [Company X] as a full-time summer intern. In my first clinically relevant position, I took my experience seriously and wanted to soak up as much as possible... [Company X] provided me the necessary foundation for understanding clinical trials and their greater role in medicine. After my internship was complete, my supervisor offered me a position working remotely as a Safety Coordinator in the Drug and Device Safety Compliance and Coding Department. At only 20 years of age, I was the youngest Safety Coordinator in their history of the company’ (001)* *‘Because of my interest in physical fitness and the human body, I enrolled in a 6 week personal training program. Each week consisted of a classroom lecture followed by a practical lab in the local gym. After passing both the written and practical exams, I completed a 30-hour internship...where I shadowed a personal trainer, witnessed multiple fitness assessments, and began designing workout programs for clients. Now as a certified trainer, I have two clients whom I instruct several times a week.’ (010)* *‘Working with children of all ages, genders, personalities, and abilities allowed me to finely tune perhaps dozens of coaching and mentoring techniques. The extraordinary number of hours spent coaching and planning team events refined my time management and organization skills.’ (016)*
Altruism	*‘... I was shocked by the amount of homeless individuals wandering the streets. I was quickly inspired to help out at one of the shelters downtown and still serve meals there whenever I can, on weekends or in the morning before classes.’ (011)* *‘...use special equipment and continual assistance to allow clients with various physical and mental impairments to ski for a half-day... there are few things as rewarding as making someone else’s day, particularly when that someone has significantly harder days than I can begin to imagine.’ (016)* *‘After his mission, the Jamaican team would not take him back; poverty left him with little future... He needed somewhere to stay and someone to guide him through his schooling. I volunteered and have taken classes with him, tutored him, corrected his papers, taught him grammar, given rides, and managed finances between him and his benefactor.’ (018)*
Entrepreneurship	*‘This year I cofounded an organization...which links refugees and recent immigrants in the... area with undergraduate students in order to decrease healthcare disparities for limited-English speaking populations.’ (006)* *‘I developed an English curriculum focused on real-life conversational skills that non-native speakers need to have in order to confidently communicate... I have taught around 15 students and am currently in the process of expanding. By the end of the summer I plan to hire two other teachers and have 30 active students.’ (014)* *‘My number one goal was to modernize legacy processes, equipment and production. Although quite capable, the squadron had become stagnant and institutionally resistant to change. I knew [I] needed to empower each of the stake holders in the change process if I were to have any chance of success... By the end of my tenure, we had jointly accomplished about 90 percent of the items on that list. The improvement was palpable.’ (013)*
Perseverance	*‘I was placed on academic probation and academic suspension... I was 17 years old, thousands of miles from home and unprepared to attend college. I withdrew from college after one more semester. I would go on to earn four degrees and ultimately a doctorate degree from [another prestigious university] with a 3.82 cumulative GPA.’ (009)* *‘A challenging but rewarding aspect of research is the importance of persistence. I struggled for six months to sequence a difficult portion of the hepatitis C genome and ultimately found a method to replicate and uncover the sequence.’ (015)*
Passion	*‘My EMS career, which had begun with a gut feeling and a friend’s recommendation, quickly grew into a passion and a sense of identity for me. By my senior year I was my college squad’s equipment officer, and was also responsible for organizing training exercises. As for that simple invitation to ride on an ambulance, well that blossomed into two part-time jobs in EMS.’ (017)* *‘For the past year I have been building a 1965 Shelby Cobra kit car that I purchased ... The process has been laborious but highly rewarding and is a dream come true. I first heard about the company in 2004 and saved money for years to be able to purchase my own kit.’ (009)*
Wisdom	*‘In food preparation, I developed the ability to take ownership of my mistakes. Work ethic and trustworthiness are very important to employers as well as patients. Working in food service for four years taught me a lot about patience and empathy.’ (003)* *‘I learned a valuable lesson from the camp participants, which was patience... Working with these youngsters not only allowed the opportunity to pass my knowledge onto future baseball players, but also it taught me how to effectively communicate with a less than receptive audience.’ (010)* *‘Work at [Company Y] has increased my ability to handle stressful situations. Building off of this skill, I have developed more personal methods that allow me to learn from stress in a healthy manner rather than just coping with it. In other words, I try to turn distress into eustress.’ (017)*
Teamwork	*‘...this experience has emphasized the importance of discipline, teamwork, and adaptability. I have learned that personal accountability allows for cooperation among members that facilitates effective patient care.’ (015)* *‘Teachers are a part of a larger education system and have to adapt to playing various roles, so acquiring teamwork and leadership skills has been vital to my teaching career.’ (021)* *‘I coordinated training between the [military’s] ships and west-coast [special forces] teams. With limited finances, this job has taught me to be resourceful... The job necessitates the cultivation of work relationships with a wide array of professionals.’ (012)*

## Discussion

The AAMC advocates for a greater emphasis on personal characteristics within a holistic approach to selection of medical students ( Association of American Medical Colleges, (2021c)), yet challenges facing medical schools to rely more on personal characteristics and less on uGPAs and MCAT scores for admission include self-interest, inertia, and philosophical factors ( Albanese *et al.,* 2003). This study demonstrates that the AMCAS application could be utilized as a tool for identifying personal characteristics that are associated with both medical school success and exceptional performance. We identified seven such characteristics using qualitative methods to assess actual student statements of exceptional performers when they applied to medical school: success in a practiced activity, altruism, entrepreneurship, perseverance, passion, wisdom and teamwork. These characteristics of exceptional performing medical students are consistent with expertise theories and support the AAMC’s core personal competencies to be successful in medical school ( Association of American Medical Colleges, (2021a)).

Deliberate practice theory describes certain features which are required to achieve expertise: persistent desire to do better, goal oriented, full concentration and effort, seeking out challenge, and reflective feedback ( Anders Ericsson, 2008). Expert performers refine their knowledge and skills by seeking out training situations that exceed their current level of reliable performance and allow them to attain desired goals ( Anders Ericsson, 2008). The concept of pursuing continuous improvement, taking initiative, and seeking innovation (features of our theme of entrepreneurship) are attributes that lead one to take deliberate efforts to change aspects of performance for the purpose to get better. Demonstrating one’s ability to engage in practice which results in improvement and success (success in a practice activity) suggests that such applicants may stick with the rigors of medical school and excel. Further, the ability for personal growth through self-discovery and awareness (wisdom) opens one up to reflective feedback. Reflective feedback is a core element of deliberate practice that involves self-monitoring and self-evaluation in order to effectively improve performance. Of note, our exceptional performers did not have exceptional MCAT scores or uGPAs with several taking the MCAT more than once( Association of American Medical Colleges, (2016)).

Other research into exceptional performance associates the personality characteristics of passion and perseverance, when combined with the pursuit of a long term goal, with successful outcomes ( Duckworth *et al.,* 2007). Defined as grit, research found that individuals with more grit have higher educational attainment ( Duckworth *et al.,* 2007) and better medical school performance ( Miller-Matero *et al.,* 2018). Because medical education entails maintaining long term effort and interest in the face of adversity (long hours, intense study, burnout), it makes sense that those who possess the ability to persevere and maintain passion would be less susceptible to burnout ( Jumat *et al.,* 2020) and that passion and perseverance manifest as personal characteristics displayed by exceptional performers in medical school.

The AAMC also reports nine core personal competencies that they consider significant to be successful in medical school ( Association of American Medical Colleges, (2021a); Koenig *et al.,* 2013). We found that several of our themes relate to these competencies. For example, the theme of altruism aligns with the competency of service orientation, both defined as “a desire to help others” and “to alleviate other’s distress” ( Association of American Medical Colleges, (2021a)). Meanwhile, the competency of resilience and adaptability aligns with the theme of perseverance - persistence under difficult situations and the ability to recover from setbacks. Our theme of entrepreneurship refers to taking initiative to create new concepts and engage in continuous improvement, similar to the competency of capacity for improvement. Lastly, both our findings and the AAMC competencies acknowledge the value of teamwork.

A core principle of the AAMCs holistic review is that selection criteria is “supported by student performance data that show that certain experiences or characteristics are linked to that individual’s likelihood of success as a student and/or physician” ( Association of American Medical Colleges, (2021c)). Given the above relationships, our themes provide empirical support that several of the AAMC’s personal competencies are not only related to a student’s likelihood of success, but are also associated with exceptionally performing medical students. Knowing that one can identify early in the admission’s process personal characteristics that may be related to medical student success can help medical school admission committees to select who to interview and can build a strong and talented medical school class. Detecting and recruiting medical student applicants with these characteristics is especially important to residency programs that attempt to recruit the best medical students ( Boyse *et al.,* 2002; Alterman *et al.,* 2011; Bhat *et al.,* 2015; Bowe, Laury and Gray, 2017; Thompson *et al.,* 2017; Agarwal *et al.,* 2018). A focus on such personal characteristics not only supports a holistic approach to selection, but has the potential to shape a physician workforce that provides society the best that medicine has to offer.

Our study has several limitations. Because the USU is a school for physicians that will enter military or public health service, prior military service is weighted favorably during the admission process. In fact, a third of our exceptional performers had prior military experience, consistent with the 37% of prior military students that matriculate to the USU. Although we attempted to generalize our findings by using honor societies (AΩA and GHHS) that represent national commonly used standards for identifying exceptional performance, we do acknowledge selection to these societies at USU and elsewhere may be impacted by bias ( Boatright *et al.,* 2017; Wijesekera *et al.,* 2019). Second, applicants may have had help in writing their statements and those statements may not reflect the applicant’s voice. Further, the AAMC promotes applicants to demonstrate the core AAMC competencies within their applications ( Duckworth *et al.,* 2007), which could persuade the applicants written responses and result in applications that reflect their ability to write convincing narratives. However, students are unlikely to possess all core competencies and those competencies they do possess are more likely to be emphasized within the application, which is true for all matriculates that applied to medical school. A third limitation of this study is that we have a lack of a comparison group (average or low performers) and therefore cannot conclude that our characteristics are unique to exceptional performers. However, causation was not the intent of the current study. Instead, we were interested to understand what characteristics were present within the AMCAS applications of exceptional performers. Future research should explore whether these or different characteristics are present in average or low performing medical student applications to further elucidate the significance of our research.

## Conclusion

Recruiting medical school applicants that have the best potential for exceptional performance is especially important in medicine because of the rigor required to complete medical training. Indeed, building a cohort of exceptional performers in medicine ultimately benefits society through enhancing the physician workforce. While empirical data clarifying what leads to exceptional performing students is lacking, this study does contribute to the literature by constructing an understanding of the personal characteristics of exceptional student performers at the entrance to medical school. These characteristics further align with both features of expertise theory and competencies deemed necessary to be successful in medical school. Our research also illustrates that it appears possible to use the AMCAS application as a tool for admission committees to assess for these personal characteristics. Future research should seek to apply these findings across a cohort of applicants at other institutions and compare characteristics between exceptional performers and either average or low performers. Although such future research is needed to determine the utility of incorporating these characteristics into the medical school admissions process, our findings offer a starting point for consideration.

## Take Home Messages

• Exceptional performing medical students display characteristics of success, altruism, entrepreneurship, perseverance, passion, wisdom and teamwork upon application to medical school.
• The American Medical College Application Service application can be used to identify personal characteristics associated with exceptional performing medical students.
• Characteristics linked to an applicant’s likelihood of success as a medical student and/or physician supports a holistic review selection process.

## Notes On Contributors

**Matthew Pflipsen** is a student in the Masters of Health Professions Education program and assistant professor in the Department of Family Medicine at the Uniformed Services University of the Health Sciences, Bethesda, Maryland. ORCID ID: https://orcid.org/0000-0003-4195-2799

**Dario Torre** is professor, Department of Medicine, Center for Health Professions Education, Uniformed Services University of the Health Sciences, Bethesda, Maryland. ORCID ID: https://orcid.org/0000-0002-4924-4888

**Steven Durning** is professor and vice chair, Department of Medicine, and director, Center for Health Professions Education, Uniformed Services University of the Health Sciences, Bethesda, Maryland. ORCID ID: https://orcid.org/0000-0001-5223-1597

## Declarations

The views expressed in this article are those of the authors and do not reflect the official policy or position of the Department of the Army, Department of the Navy, Department of the Air Force, Uniformed Services University of the Health Sciences, Department of Defense, or the U.S. government.

## References

[ref1] Agarwal V. Bump G. M. Heller M. T. Chen L. W. (2018) Do Residency Selection Factors Predict Radiology Resident Performance? Acad Radiol. 25(3), pp.397–402. 10.1016/j.acra.2017.09.020 29239834

[ref2] Albanese M. A. Snow M. H. Skochelak S. E. Huggett K. N. (2003) Assessing personal qualities in medical school admissions. Academic Medicine. 78(3), pp.313–321. 10.1097/00001888-200303000-00016 12634215

[ref3] Alpha Omega Alpha Honor Medical Society , (2021): Constitution. Available at: https://alphaomegaalpha.org/pdfs/2020revConstitution.pdf( Accessed: 13/01/2021).

[ref4] Alterman D. M. Jones T. M. Heidel R. E. Daley B. J. (2011) The predictive value of general surgery application data for future resident performance. J Surg Educ. 68(6), pp.513–518. 10.1016/j.jsurg.2011.07.007 22000538

[ref5] Anders Ericsson K. (2008) Deliberate Practice and Acquisition of Expert Performance: A General Overview. Academic Emergency Medicine. 15(11), pp.988–994. 10.1111/j.1553-2712.2008.00227.x 18778378

[ref6] Arnold P. Gold Foundation . (2021a): Gold Humanism Honor Society Chapter Toolkit. Available at: https://s3.amazonaws.com/gold-foundation/wp-content/uploads/2019/02/2019-Toolkit-FINAL.pdf( Accessed: 13/01/2021).

[ref7] Arnold P. Gold Foundation . (2021b): Gold Humanism Honor Society Chapter List. Available at: https://www.gold-foundation.org/programs/ghhs/chapter-list/( Accessed: 13/01/2021).

[ref8] Association of American Medical Colleges , (2016): FACTS Table A-16: MCAT Scores and GPAs for Applicants and Matriculants to U.S. Medical Schools, 2006-2007 through 2015-2016.

[ref9] Association of American Medical Colleges , (2021a): The Core Competencies for Entering Medical Students. Available at: https://students-residents.aamc.org/applying-medical-school/article/core-competencies( Accessed: 10/01/2021).

[ref10] Association of American Medical Colleges , (2021b): How to Apply to Medical School with AMCAS: Section 5 of the AMCAS Application: Work and Activities. Available at: https://students-residents.aamc.org/applying-medical-school/article/section-5-work-and-activities( Accessed: 13/01/2021).

[ref11] Association of American Medical Colleges , (2021c): Holistic Review: Core Principles. Available at: https://www.aamc.org/services/member-capacity-building/holistic-review( Accessed: 13/01/2021).

[ref12] Bhat R. Takenaka K. Levine B. Goyal N. (2015) Predictors of a Top Performer During Emergency Medicine Residency. J Emerg Med. 49(4), pp.505–512. 10.1016/j.jemermed.2015.05.035 26242925

[ref13] Boatright D. Ross D. O’Connor P. Moore E. (2017) Racial Disparities in Medical Student Membership in the Alpha Omega Alpha Honor Society. JAMA internal medicine. 177(5), pp.659–665. 10.1001/jamainternmed.2016.9623 28264091 PMC5818775

[ref14] Bowe S. N. Laury A. M. and Gray S. T. (2017) Associations between Otolaryngology Applicant Characteristics and Future Performance in Residency or Practice: A Systematic Review. Otolaryngol Head Neck Surg. 156(6), pp.1011–1017. 10.1177/0194599817698430 28349776

[ref15] Boyatzis R. E. (1998) Transforming qualitative information: Thematic analysis and code development. Thousand Oaks, CA, US: Sage Publications, Inc.

[ref16] Boyse T. D. Patterson S. K. Cohan R. H. Korobkin M. (2002) Does medical school performance predict radiology resident performance. Acad Radiol. 9(4), pp.437–445. 10.1016/s1076-6332(03)80189-7 11942658

[ref17] Braun V. and Clarke V. (2006) Using thematic analysis in psychology. Qualitative Research in Psychology. 3(2), pp.77–101. 10.1191/1478088706qp063oa

[ref18] Busche K. Elks M. L. Hanson J. T. Jackson-Williams L. (2020) The Validity of Scores From the New MCAT Exam in Predicting Student Performance: Results From a Multisite Study. Acad Med. 95(3), pp.387–395. 10.1097/acm.0000000000002942 31425189

[ref19] Cortez A. R. Winer L. K. Kim Y. Hanseman D. J. (2019) Predictors of medical student success on the surgery clerkship. Am J Surg. 217(1), pp.169–174. 10.1016/j.amjsurg.2018.09.021 30266418

[ref20] Donnon T. Paolucci E. O. and Violato C. (2007) The predictive validity of the MCAT for medical school performance and medical board licensing examinations: a meta-analysis of the published research. Acad Med. 82(1), pp.100–106. 10.1097/01.ACM.0000249878.25186.b7 17198300

[ref21] Duckworth A. L. Peterson C. Matthews M. D. and Kelly D. R. (2007) Grit: Perseverance and Passion for Long-Term Goals. Journal of Personality and Social Psychology. 92(6), pp.1087–1101. 10.1037/0022-3514.92.6.1087 17547490

[ref22] Duvivier R. J. van Dalen J. Muijtjens A. M. Moulaert V. R. (2011) The role of deliberate practice in the acquisition of clinical skills. BMC Med Educ. 11, p.101. 10.1186/1472-6920-11-101 22141427 PMC3293754

[ref23] Ericsson K. A. (2015) Acquisition and Maintenance of Medical Expertise: A Perspective From the Expert-Performance Approach With Deliberate Practice. Academic Medicine. 90(11), pp.1471–1486. 10.1097/ACM.0000000000000939 26375267

[ref24] Furstenberg S. and Harendza S. (2017) Differences between medical student and faculty perceptions of the competencies needed for the first year of residency. BMC Med Educ. 17(1), p.198. 10.1186/s12909-017-1036-7 29121897 PMC5680802

[ref25] Haight S. J. Chibnall J. T. Schindler D. L. and Slavin S. J. (2012) Associations of medical student personality and health/wellness characteristics with their medical school performance across the curriculum. Acad Med. 87(4), pp.476–485. 10.1097/ACM.0b013e318248e9d0 22361792

[ref26] Herrera L. N. Khodadadi R. Schmit E. Willig J. (2019) Which Student Characteristics Are Most Important in Determining Clinical Honors in Clerkships? A Teaching Ward Attending Perspective. Acad Med. 10.1097/acm.0000000000002836 31192796

[ref27] Hojat M. Erdmann J. B. and Gonnella J. S. (2013) Personality assessments and outcomes in medical education and the practice of medicine: AMEE Guide No. 79. Med Teach. 35(7), pp. e1267–e1301. 10.3109/0142159x.2013.785654 23614402

[ref28] Jumat M. R. Chow P. K.-H. Allen J. C. Lai S. H. (2020) Grit protects medical students from burnout: a longitudinal study. BMC medical education. 20(1), pp.1–9. 10.1186/s12909-020-02187-1 PMC7425562 32787919

[ref29] Kiger M. E. and Varpio L. (2020) Thematic analysis of qualitative data: AMEE Guide No. 131. Med Teach.pp.1–9. 10.1080/0142159x.2020.1755030 32356468

[ref30] Koenig T. W. Parrish S. K. Terregino C. A. Williams J. P. (2013) Core Personal Competencies Important to Entering Students’ Success in Medical School: What Are They and How Could They Be Assessed Early in the Admission Process? ACADEMIC MEDICINE. 88(5), pp.603–613. 10.1097/ACM.0b013e31828b3389 23524928

[ref31] Lievens F. Ones D. S. and Dilchert S. (2009) Personality scale validities increase throughout medical school. J Appl Psychol. 94(6), pp.1514–1535. 10.1037/a0016137 19916659

[ref32] Miller-Matero, L. R., Martinez, S., MacLean, L., Yaremchuk, K., *et al.*(2018) Grit: A predictor of medical student performance. Educ Health (Abingdon). 31(2), pp.109–113. 10.4103/efh.EfH_152_16 30531053

[ref33] Monroe A. Quinn E. Samuelson W. Dunleavy D. M. (2013) An overview of the medical school admission process and use of applicant data in decision making: what has changed since the 1980s? Acad Med. 88(5), pp.672–681. 10.1097/ACM.0b013e31828bf252 23524917

[ref34] Moulaert V. Verwijnen M. G. Rikers R. and Scherpbier A. J. (2004) The effects of deliberate practice in undergraduate medical education. Med Educ. 38(10), pp.1044–1052. 10.1111/j.1365-2929.2004.01954.x 15461649

[ref35] Plant E. A. Ericsson K. A. Hill L. and Asberg K. (2005) Why study time does not predict grade point average across college students: Implications of deliberate practice for academic performance. Contemporary Educational Psychology. 30(1), pp.96–116. 10.1016/j.cedpsych.2004.06.001

[ref36] Saguil A. Dong T. Gingerich R. J. Swygert K. (2015) Does the MCAT predict medical school and PGY-1 performance? Mil Med. 180(4 Suppl), pp.4–11. 10.7205/milmed-d-14-00550 25850120

[ref37] Thompson R. H. Lohse C. M. Husmann D. A. Leibovich B. C. (2017) Predictors of a Successful Urology Resident Using Medical Student Application Materials. Urology. 108, pp.22–28. 10.1016/j.urology.2017.06.046 28751165

[ref38] Varpio L. Ajjawi R. Monrouxe L. V. O’Brien B. C. (2017) Shedding the cobra effect: problematising thematic emergence, triangulation, saturation and member checking. Med Educ. 51(1), pp.40–50. 10.1111/medu.13124 27981658

[ref39] White C. B. Dey E. L. and Fantone J. C. (2009) Analysis of factors that predict clinical performance in medical school. Adv Health Sci Educ Theory Pract. 14(4), pp.455–464. 10.1007/s10459-007-9088-9 18030590

[ref40] Wijesekera T. P. Kim M. Moore E. Z. Sorenson O. (2019) All Other Things Being Equal: Exploring Racial and Gender Disparities in Medical School Honor Society Induction. Acad Med. 94(4), pp.562–569. 10.1097/acm.0000000000002463 30234509

