# Supplementary material for: Beyond Grade Point Averages and Medical College Admission Test Scores: A Thematic Analysis of Exceptional Performing Medical Student Applications
Source: MedEdPublish (2016). 2021 Jun 2;10:151. [Version 1] doi: 10.15694/mep.2021.000151.1 (PMC10939559; doi:10.15694/mep.2021.000151.1)
Supplement: Peer review history

# Reviewer report: Reviewer response for version 1

**DOI:** 10.21956/mep.18973.r27176

**Published:** 2021-10-06

**Authors:** P Ravi Shankar

**Affiliations:** 1 American International Medical University

This review has been migrated. The reviewer awarded 4 stars out of 5 I read this article with great interest. Selecting students for medical school has always been an area of interest for me. The process described by the authors is mainly in the United States context though similar parameters are also used in other countries. The limitations section is comprehensive, and the authors provide insights into the generalizability of their study. Some countries continue to select students based only on previous academic performance or ranks in an entrance examination while others are now also considering other characteristics. Selecting doctors who will serve in rural and underserved areas is becoming important. Many schools also are using multiple mini interviews as a part of student selection. Predicting which students are likely to graduate and graduate and perform exceptionally in practice is important for medical educators. All medical educators will find this article to be of interest.

Reviewer Expertise:

NA

No decision status is available

# Reviewer report: Reviewer response for version 1

**DOI:** 10.21956/mep.18973.r27175

**Published:** 2021-10-04

**Authors:** Leila Niemi-Murola

**Affiliations:** 1 University of Helsinki

This review has been migrated. The reviewer awarded 4 stars out of 5 This is an interesting article focusing on medical college admission scores. Traditionally, high scores in admission test have granted acceptance. Medical schools strive to accept talented students, but there may other means to find the exceptionally performing ones. The authors have performed a qualitative study in order to identify themes in medical college applications that might reflect the characteristics of exceptional performing medical students. The applications were explored from a constructivist paradigm using thematic analysis.The authors give a detailed description of the methodology, and the theoretical part is highly appreciated. They constructed seven themes from the data: success in a practiced activity, altruism, entrepreneurship, passion, perseverance, teamwork, and wisdom.It is very likely that analysis of the applications give an interesting perspective to the personality of the applicants. The authors discuss the limitations of the study well. If this method will be accepted as an official one, it might be that the applicants will seek professional assistance when writing their applications. One solution to this might be that this paper will be included in the test. The authors’ wish for a holistic selection process seems to be well grounded.

Reviewer Expertise:

NA

No decision status is available

# Reviewer report: Reviewer response for version 1

**DOI:** 10.21956/mep.18973.r27177

**Published:** 2021-06-02

**Authors:** Aaron Saguil

This review has been migrated. The reviewer awarded 4 stars out of 5 Thank you for providing me the opportunity to review this paper. The question of what factors best predict success in medical school is evergreen, and this paper represents an important contribution to the field.The authors’ novel approach of taking medical students with demonstrated exceptional performance, as measured by inclusion in AOA and GHHS, and then looking retrospectively as their medical school applications adds to the field. Linking the performance predictors from their thematic analysis of records (success in a practiced activity, altruism, entrepreneurship, passion, perseverance, teamwork, and wisdom) to Ericsson’s deliberate practice theory, Duckworth’s grit, and the AAMC entering competencies shows that their findings have strong theoretical underpinnings.My greatest concerns with this piece were adequately addressed in the authors’ limitations section. There is literature to suggest that AOA is not without inherent selection bias, which potentially calls the results of this study into question. Additionally, there is no comparator group—it could be that average or even low-performing students have the same exceptional performance predictors to the same degree. The authors acknowledge this, and call for additional research to investigate this further. I hope they choose to do it.I recommend this paper for publication—it is a useful addition to the admissions literature and promotes holistic review practices.

Reviewer Expertise:

NA

No decision status is available
